# Oxidative Stress Is Related to the Deleterious Effects of Heme Oxygenase-1 in an *In Vivo* Neuroinflammatory Rat Model

**DOI:** 10.1155/2013/264935

**Published:** 2013-03-05

**Authors:** Claire Tronel, Gaël Y. Rochefort, Nicolas Arlicot, Sylvie Bodard, Sylvie Chalon, Daniel Antier

**Affiliations:** ^1^UMR INSERM U930, Université François Rabelais de Tours, PRES Centre Val de Loire Université, 37000 Tours, France; ^2^EA 4708, IPROS, CHR Orléans, BP 2439, 1 rue Porte Madeleine, 45032 Orléans, France; ^3^Département Pharmacie, CHRU de Tours, Tours, France

## Abstract

Heme oxygenase-1 (HO-1) induction is associated with beneficial or deleterious effects depending on the experimental conditions adopted and the neurodegenerative rodent models used. The present study aimed first to evaluate the effects of cerebral HO-1 induction in an *in vivo* rat model of neuroinflammation by intrastriatal injection of quinolinic acid (QA) and secondly to explore the role played by reactive oxygen species (ROS) and free iron (Fe^2+^) derived from heme catabolism promoted by HO-1. Chronic I.P. treatment with the HO-1 inductor and substrate hemin was responsible for a significant dose-related increase of cerebral HO-1 production. Brain tissue loss, microglial activation, and neuronal death were significantly higher in rats receiving QA plus hemin (H-QA) versus QA and controls. Significant increase of ROS production in H-QA rat brain was inhibited by the specific HO-1 inhibitor ZnPP which supports the idea that ROS level augmentation in hemin-treated animals is a direct consequence of HO-1 induction. The cerebral tissue loss and ROS level in hemin-treated rats receiving the iron chelator deferoxamine were significantly decreased, demonstrating the involvement of Fe^2+^in brain ROS production. Therefore, the deleterious effects of HO-1 expression in this *in vivo* neuroinflammatory model were linked to a hyperproduction of ROS, itself promoted by free iron liberation.

## 1. Introduction

Neuroinflammation is well known as an important element of brain disorders and in particular neurodegenerative diseases [1]. Microglial cells play a role often compared to the macrophage function in the central nervous system [2, 3]. Quiescent microglia are activated in the occurrence of brain damage such as oxidative stress, leading to an inflammatory cascade response. This activation is characterized by morphologic modifications and mostly secretion of proinflammatory factors such as cytokines or reactive oxygen species (ROS) [4, 5]. Without appropriate regulation, those proinflammatory agents cause brain damage to worsen. Therefore neuroinflammation is a relevant target on purpose to treat neurodegenerative diseases. Many experimental studies have been carried out to explore the effects of anti-inflammatory treatments against neurodegeneration especially in animal models, demonstrating that treatment with nonsteroidal anti-inflammatory (NSAIDs) drugs [6–8] or nitric-oxide-releasing NSAIDs [9] could have a beneficial effect on neurodegenerative diseases such as Alzheimer's disease (AD), Parkinson's disease, or multiple sclerosis. However, prospective anti-inflammatory strategies against disease progression in human subjects with established AD have failed to show significant positive results [10].

Heme oxygenase (HO) is the final enzyme involved in the degradation of heme [11]. The inducible isoform HO-1 has been implicated in the regulation of inflammation and this enzyme is overexpressed in response to different stimuli such as oxidative and nitrosative stresses [12]. The induction of HO-1 increases the heme catabolism into biliverdin and bilirubin, potent antioxidant scavenging peroxy radicals, and inhibits lipid peroxidation [13]. 

Previous studies have reported controversial effects of HO-1 induction, either deleterious or beneficial depending on the different neuroinflammatory models and various drug exposure methods. For example, in microglial cell cultures, HO-1 induction has been shown as protective in a model of neurotoxicity induced by glutamate [14]. In experimental autoimmune encephalomyelitis, HO-1 induction demonstrated a protective role by inhibiting major histocompatibility complex II expression and lymphocyte proliferation [15]. Conversely, HO-1 induction was associated with a deleterious iron accumulation in cultured astrocytes [16] as well as in activated microglia in a rodent stroke model [17]. The anti-inflammatory role of the products of heme degradation and the potential activation of HO-1 in the brain support the potential interest of this enzyme in neuroinflammation treatment. We hypothesized that deleterious HO-1 activation effects are linked to products resulting from heme degradation during brain inflammation.

In order to test this hypothesis, we experimentally increased the expression of HO-1 using the specific inducer hemin, in a rat model of neuroinflammation obtained by unilateral striatal injection of quinolinic acid (QA). This well-known model of Huntington's diseases [18] has recently been shown to be useful to study the overexpression of the translocator protein (TSPO) as a relevant marker of neuroinflammation [19]. QA is a strong agonist of glutamate NMDA (*N*-methyl-*D*-aspartate) receptors. Overactivation of NMDA receptors causes a massive intracellular influx of calcium that leads to neuronal death by activation of various enzymes (lipases, proteases, endonucleases) triggering different cell components then leading to neuronal death [20]. Factors released during the death of neurons rapidly lead to an important microglial activation.

Therefore, the purpose of the present study was first to assess *in vivo *the effects of the HO-1 inducer hemin on both neuronal survival and microglial activation in the neuroinflammatory excitotoxic rat model induced using QA intrastriatal injection and secondly to investigate the underlying mechanisms, especially focusing on ROS production in brain structures and the hypothetic role of iron derived from HO-1 enzymatic activity.

## 2. Materials and Methods

### 2.1. Animals

Sixty-six male Wistar rats (Janvier, l'Arbresle, France) weighing ~340 ±10 g were used. The experiments were performed in accordance with the Guideline for the Care and Use of Laboratory Animals published by the US National Institutes of Health (NIH Publication no. 85-23, revised 1996) and with European Directives (86/609/CEE) and approved by local ethical committee (Agreement no. 2012-03-1). Rats were kept in a temperature (23 ± 0.5°C)—and humidity (43 ± 8%)—controlled environment under a 12 h light-dark cycle with food and water available ad libitum. All efforts were made to minimize animal suffering and discomfort.

### 2.2. Determination of the Relevant Hemin Dose

We first investigated the potential dose-related effect of chronic intraperitoneal (I.P.) treatment with hemin on cerebral expression levels of HO-1. Based on a previously published study [21], rats received a hemin solution in a daily dose of 10 mg/kg (*n* = 3) or 50 mg/kg (*n* = 3) over a 4-day period (final volume of 100 *μ*L by injection, in DMSO). Hemin-treated rats were compared to a control group receiving its vehicle DMSO (100 *μ*L; daily I.P.; *n* = 3). The DMSO solvent is known to be a strong antioxidant compound [22] but the volume (100 *μ*L) necessary to solubilize hemin for I.P. injection has been already used in previous studies without influence on biological parameters and the anti-inflammatory properties of DMSO reported in the literature considered large amounts of DMSO up to 6 mL/kg [23]. On day 5, the animals were euthanatized by decapitation and their brains were removed to analyze HO-1 expression by western blotting (WB).

The entire right hemispheres were rapidly crushed by Turrax, homogenized with lysis buffer 1% sodium deoxycholate, 0.1% SDS, 1% tritonX-100, 10 mM Tris-HCl (pH 8.0), 150 mM NaCl, and an inhibitor protease cocktail (chymotrypsin, thermolysin papain, pronase, pancreatic extract and trypsin inhibitor; Roche), and centrifuged at 20,000 g for 1 hour. After denaturation by boiling (100°C, 5 min), beta mercaptoethanol and bromophenol blue were added to samples. Protein quantification was performed according to the Bradford method and then 25 mg of protein were separated on a 10% polyacrylamide gel and were transferred (30 min, 110 V) onto a nitrocellulose membrane (Amersham). Blots were blocked for 2 hours at room temperature with 5% (v/v) nonfat dried milk in Tris-buffered saline [10 mM Tris-HCl (pH 8.0) and 150 mM NaCl] containing 0.05% Tween 20. The membrane was incubated overnight at 4°C with rabbit polyclonal antibody against HO-1 (1/400, Bio-Rad, Marnes-la-Coquette, France) or with a mouse polyclonal antibody against rat beta-3 tubulin used as housekeeping protein (1/400, Tubb3, Santa Cruz Biotechnologies, CA, USA). The blot was then incubated with the conjugated goat antirabbit or antimouse horseradish peroxidase (1/1000, BioSource) for 2 h at room temperature. Immunoreactive proteins were detected with the ECL western blotting detection system (Amersham) using an imager (Gel doc XRS +, Biorad, Marnes-la-Coquette, France). The results were analyzed with Quantity One (Biorad, Marnes-la-Coquette, France).

### 2.3. Excitotoxic Neuroinflammation Model

One day after the first I.P. injection of hemin or its vehicle, the rats were anesthetized with isoflurane (4% for anesthesia induction and thereafter 2% for its maintenance) and placed in a stereotaxic David Kopf apparatus (tooth bar: −3.3 mm). The animals were unilaterally injected with 150 nmol of quinolinic acid (QA) (Sigma-Aldrich, Lyon, France) or its vehicle (0.1 M PBS, pH 7.4) into the right striatum (injection rate: 0.5 *μ*L/min) using a 25-*μ*L microsyringe (Hamilton, Bonaduz, Switzerland) and a micropump (KD Scientific, Holliston, MA, USA). Two microliters of QA were injected at the following coordinates: AP: +0.7 mm; ML: −3 mm; DV: −5.5 mm from bregma, according to Paxinos and Watson [24]. Body temperature (36.9 ± 0.6°C) was monitored during the surgery with a thermal probe. The injection syringe was left in place for additional 4 min to avoid QA backflow and then slowly removed. The scalp was sutured and animals replaced in their cages and examined daily until sacrifice.

### 2.4. Experimental Procedure and Drug Treatment

Drugs or vehicles for control were administered I.P. the day before surgery, 2 h before surgery, and daily for 2 days. Consecutively to the above study (see Section 3), rats treated by hemin received 50 mg/kg I.P.

#### 2.4.1. Effect of Hemin Treatment on Neuron Survival and Microglia Activation

The animals were randomly divided as follows: control group (DMSO I.P.; PBS intrastriatal; *n* = 6); QA (DMSO I.P.; QA 150 nmol/2 *μ*L intrastriatal; *n* = 6); H (hemin 50 mg/kg; PBS intrastriatal; *n* = 6); H-QA (hemin 50 mg/kg I.P.; QA 150 nmol/2 *μ*L intrastriatal; *n* = 6). At day 3 after surgery, the rats were euthanized for immunohistochemistry (IHC) processing. The rats were deeply anesthetized by I.P. injection of pentobarbital (Céva Santé Animale, Paris, France), perfused through the heart with 250 mL of heparinized (Héparine Choay, Sanofi-Aventis, Vitry-sur-Seine, France) saline (1 UI/mL of saline), and then followed by 400 mL of 4% paraformaldehyde (PFA, Sigma-Aldrich, Lyon, France). The brains were removed and fixed in 4% PFA for 2 h then stored 48 h in 30% sucrose and frozen at −80°C.

Five transversal sections 40 *μ*m thick of both the striatum and hippocampus regions were used for IHC staining of neurons or activated microglia. Endogenous peroxidase was blocked using 3% H_2_O_2_ in 10% methanol and distilled water for 15 min. Slices were incubated overnight at room temperature with primary antibodies 1 : 500 diluted NeuN (Millipore, Molsheim, France) or 1 : 500-diluted Ox-42 (AbD Serotec, Düsseldorf, Germany), 0.1 M PBS supplemented with 0.2% v/v of Tween and 2% v/v of normal horse serum. As we used horse secondary antibodies, nonspecific binding sites were blocked by adding 2% horse serum and then incubated simultaneously with primary antibodies overnight at room temperature. Sections were then incubated with biotinylated horse anti-mouse IgG secondary antibodies (AbCys, Paris, France) for 90 min at room temperature. Neurons or activated microglia were visualized by staining with streptavidin-biotin-conjugated horseradish peroxidase (AbCys, Paris, France) for 60 min at room temperature. Peroxidase was developed for 3 min with diaminobenzidine at room temperature. Slices were analyzed under a light binocular microscope (Leica, Wetzlar, Germany) and histological analysis was performed with Histolab imagery software (US Histology Laboratories, Rockville, USA). Neuron survival and microglia activation were analyzed in 2 different areas for each brain: the area of QA injection (bregma +0.7 mm) and the cortical area at a distance from the site of injury (bregma −3 mm). The neurons were visually counted and the percentage of neuronal loss in the ipsi- versus contra-lateral hemisphere was calculated in matching areas of analysis. For Ox-42, the total surface area occupied by activated microglia was automatically measured by the imagery software and the percentage of increase in ipsi- versus contralateral hemisphere was measured for each section. Five striatal slices were made within the QA injection site (from Bregma +0.6 to Bregma +0.8). Three areas per hemisphere were then randomly selected and neurons and surface occupied by activated microglia were counted by 2 independent operators. The surface area of tissue destruction in striatum was measured using Beta-Vision Plus software (Biospace Lab, Paris, France) and the percentage of tissue loss in ipsi- versus contra-lateral hemisphere was calculated on 5 slices per animal.

#### 2.4.2. Effect of Hemin Treatment on Cerebral Reactive Oxygen Species Production

To understand the results observed in hemin-treated rats (see Section 3), a complementary study was performed focusing on the effect of HO-1 activity on ROS production in neurons of the striatum. The specificity of HO-1 influence on ROS was tested by adding a group of animals exposed to the inhibitor of HO-1 activity zinc protoporphyrin IX (ZnPP, Sigma-Aldrich) in a concentration of 50 mg/kg 24 h and 2 h before surgery [25].

Twenty rats were separated into 4 groups as follows: control group (DMSO I.P.; PBS intrastriatal; *n* = 5); QA (DMSO I.P.; QA 150 nmol/2 *μ*L intrastriatal; *n* = 5); H-QA (hemin 50 mg/kg I.P.; QA 150 nmol/2 *μ*L intrastriatal; *n* = 5); ZnPP-H-QA (ZnPP 50 mg/kg I.P.; hemin 50 mg/kg I.P.; QA 150 nmol/2 *μ*L intrastriatal; *n* = 5). Bearing in mind the short life span of ROS [26], the influence of QA and hemin on ROS expression levels was determined in the striatum at 1 h after QA injection in both QA and H-QA groups. ROS production was quantified by measuring the fluorescence of dihydroethidium (DHE, Sigma-Aldrich, Lyon, France,) which is oxidized into ethidium and 2-hydroxy ethidium by intracellular ROS [27]. After intercalation into DNA, both products derived from DHE oxidation emit a red fluorescence proportional to ROS production. Therefore, neuron immunofluorescence detection and ROS quantification were performed simultaneously to normalize ROS fluorescence measurements to the number of preserved neurons after QA injection. One hour after surgery, the animals were deeply anesthetized by I.P. injection of pentobarbital and perfused through the heart with 250 mL of heparinized saline (1 UI/mL of saline) to limit noise floor caused by blood autofluorescence [28]. ROS quantification in frozen tissue has already been described in the literature especially with brain material [29–31]. After perfusion, the brains were removed and snap-frozen at −80°C. Sections (20 *μ*m) were fixed in PFA 4% (15 min) for neuron immunostaining, before saturation of nonspecific sites with normal horse serum (1 : 200) for 30 min at room temperature and incubation with 1 : 500 diluted primary antibody NeuN (Millipore, Molsheim, France) overnight at room temperature. Revelation was made using a secondary antibody fluorescein isothiocyanate (FITC) conjugated (1 : 200, Rockland Immunochemicals, Gilbertsville, USA) for 3 h at room temperature. ROS analyzing was performed using DHE (4 *μ*M, Sigma-Aldrich, Lyon, France) applied to slides then cover-slipped before incubation (37°C, 30 min) in a dark humidified chamber. Preliminary study was performed to check out that the tissue fixation did not alter the DHE signal. We compared fluorescence in frozen versus fixed/frozen brain slices and then confirmed that the DHE signal was unchanged regardless of the fixation (unpublished data). FITC and DHE fluorescence were measured using an Olympus BX51 microscope and analyzed with Cell D (Olympus, Hamburg, Germany) to measure the intensity of fluorescence and Image J (Rasband, WS, Image J, US National Institute of Health, Bethesda, MD, USA) to manually perform neuron counting. Then, the intensity of ROS fluorescence in surviving neurons population and the ratio augmentation rate (%) in ipsi- versus the contra-lateral hemisphere was calculated.

#### 2.4.3. Influence of Ferrous Iron (Fe^2+^) Chelator Treatment on Hemin Effects

Given that ferrous iron (Fe^2+^) is a product of heme, we investigated the influence of the Fe^2+^ chelator deferoxamine (DFX) on neuron survival, microglia activation, tissue destruction, and ROS production in QA and hemin-treated rats. The compound was administered chronically according to the same schedule as hemin in 2 additional groups of animals as follows: DFX-QA (DFX, 150 mg/kg I.P.; QA 150 nmol/2 *μ*L intrastriatal; *n* = 5 for IHC) and DFX-H-QA (DFX, 150 mg/kg I.P.; hemin 50 mg/kg I.P.; QA 150 nmol/2 *μ*L intrastriatal; *n* = 5 for IHC; *n* = 3 for ROS analysis). IHC and ROS measurement were performed according to the methodology described above.

### 2.5. Statistical Analysis

Results are expressed as means ± SEM. Data for multiple variable comparisons were analyzed by a one-way ANOVA followed by a Newman-Keul's test as a posthoc test using GraphPad Prism version 5 (GraphPad Software, San Diego, CA, USA). The level of significance was *P* < 0.05.

## 3. Results

### 3.1. Influence of Chronic Hemin Treatment on HO-1 Expression in the Brain

**Figure 1:**
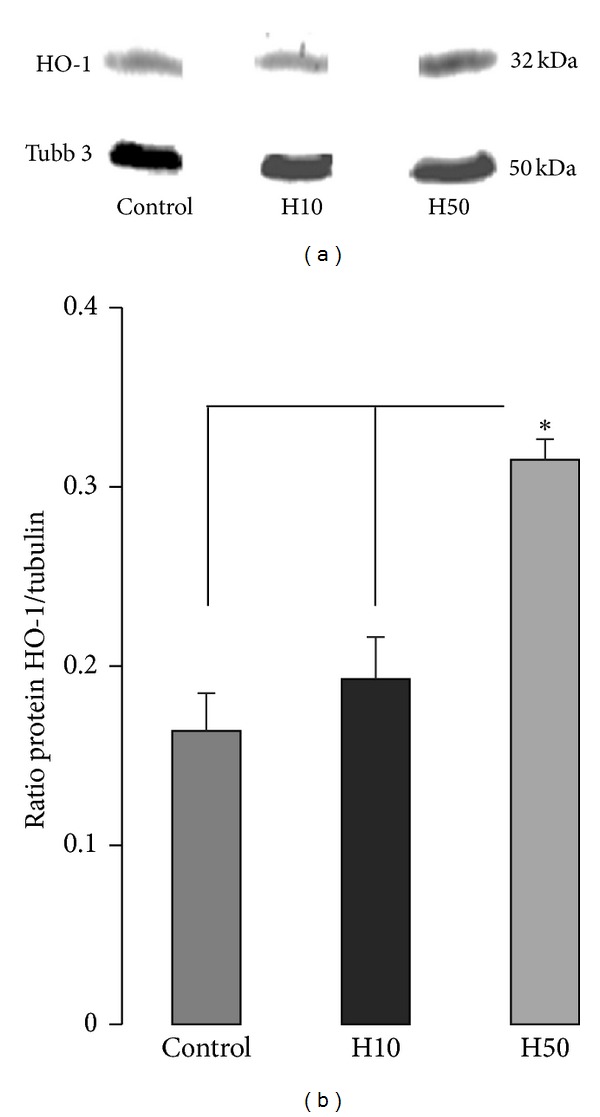
Dose effect of systemic hemin treatment on HO-1 protein expression in the brain (a) HO-1 and tubuline beta 3 (Tubb 3) western blotting bands for control (*n* = 3), hemin 10 (*n* = 3), and hemin 50 mg/kg (*n* = 3) groups. (b) DO of HO-1 normalized with Tubb-3. *P < 0.05.

This preliminary study aimed to evaluate the effect of 4-day chronic treatment with either 10 or 50 mg/kg of the HO-1 inducer hemin on HO-1 protein expression levels in the rat brain. Western blotting measurements showed a significant (*P* < 0.05) increase of HO-1 protein expression in rats exposed to 50 mg/kg hemin versus 10 mg/kg and control (Figures 1(a) and 1(b)). Results normalized with tubulin protein are illustrated in Figure 1(b). Therefore, the highest dose of hemin (50 mg/kg) was used to evaluate the influence of HO-1 induction in the neuroinflammatory *in vivo* model.

### 3.2. Effect of Hemin Treatment and Influence of Ferrous Iron Chelator DFX on Neuron Survival, Cerebral Macroscopic Integrity, and Microglial Activation

#### 3.2.1. In the Area of Quinolinic Acid Injection: Bregma +0.7 mm

**Figure 2:**
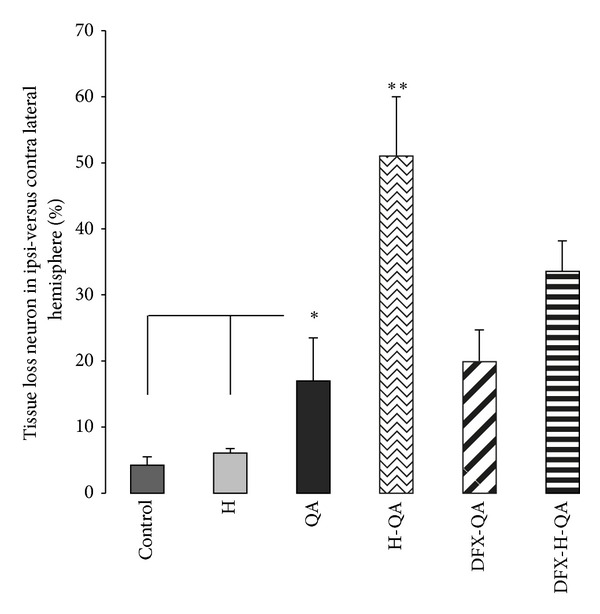
Relative cerebral tissue loss in the ipsilateral hemisphere versus the contralateral hemisphere 3 days after injury in control (*n* = 6), H (*n* = 5), QA (*n* = 6), H-QA (*n* = 5), DFX-QA (*n* = 5) and DFX-H-QA (*n* = 5) groups. *P < 0.05; **P < 0.01.

Results are illustrated as a percentage of tissue loss in the ipsilateral hemisphere for all groups in Figure 2. In QA (*n* = 6), H-QA (*n* = 5), DFX-QA, and DFX-H-QA (*n* = 5) groups, major tissue destruction within the striatum did not allow neuron survival or microglia activation analysis. We observed in the ipsilateral cerebral hemisphere exposed to QA injection a major tissue loss (17 ± 6.5%) significantly (*P* < 0.01) worsened by hemin treatment (51 ± 9.0% in H-QA animals). DFX treatment significantly (*P* < 0.05) decreased tissue loss caused by hemin (33 ± 4.6% in DFX-H-QA group) but had no effect on QA alone (20.0 ± 4.7% in DFX-QA group, *n* = 5). No cerebral tissue loss was observed, except mechanical lesions consequent to the needle injection, in control and H groups. 

**Figure 3:**
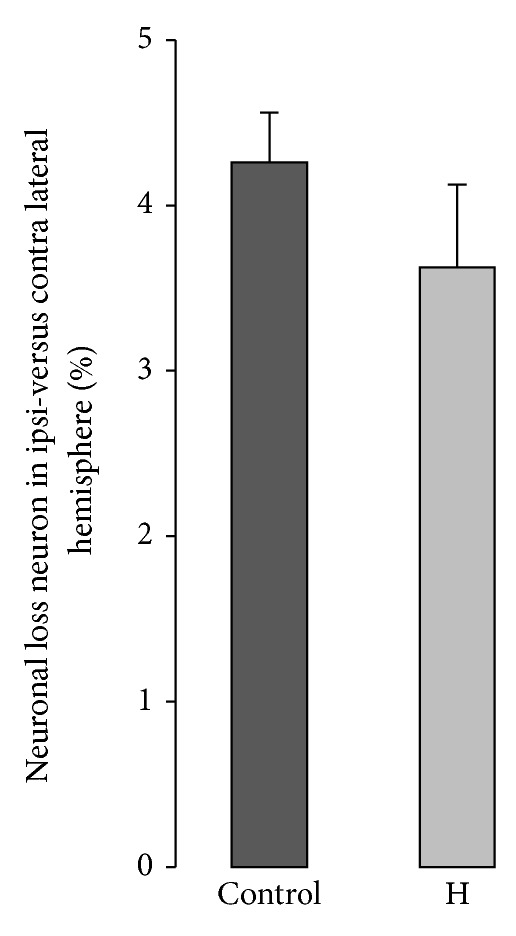
Relative neuronal loss in the ipsi- versus contra-lateral striatum in control and H groups 3 days after striatal injection.

NeuN IHC was performed by 2 independent operators. For each brain, 5 slices were processed and 3 areas in the striatum per hemisphere were used for the measurement. Neuronal loss in the ipsilateral hemisphere did not show any significant difference between H and control groups (Figure 3).

Microglial activation was analyzed using Ox-42, an antibody specific to CD11b expressed in activated microglia. The relative area of brain slices occupied by activated microglial cells was measured in 3 areas per hemisphere (5 slices) of control and H groups and the overall level in the ipsi- and contralateral hemispheres was compared. No significant difference was observed between the animals.

#### 3.2.2. In a Cortical Area Remote from the Site of Injury: Bregma −3 mm

**Figure 4:**
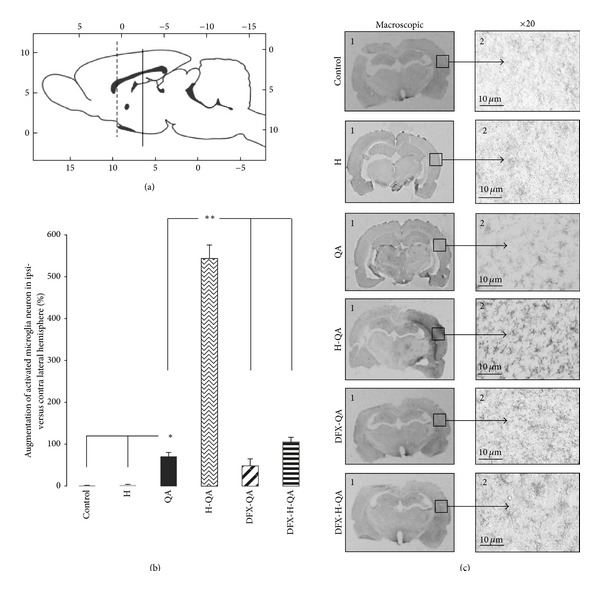
Microglial activation in the cortex (bregma −3 mm) 3 days after intrastriatal injection. (a) Sagittal rat brain representation [24]. The dotted and full lines symbolize, respectively, the site of injury (bregma +0.7 mm) and the area where Ox-42 immunohistochemistry was performed (bregma −3 mm). (b) Increasing of microglia activation in the ipsi- versus the contra-lateral hemisphere for each brain. Measurements were performed in the cortex (3 areas per hemisphere) at a distance of 3.7 mm posterior from the site of injury (bregma −3 mm). *P < 0.05; **P < 0.01. (c) Ox-42 immunochemistry in control, H, QA, H-QA, DFX-QA, and DFX-H-QA brains. 1: macroscopic view (bregma −3 mm). 2: microscopic view (×20) of activated microglia in the cortex of the ipsilateral hemisphere.

In this area remote from the site of injury (Figure 4(a)), no difference in the number of neurons was found between contra- and ipsilateral cortex, regardless of the rats treatments (data not shown). However, the augmentation rate of activated microglia, evaluated by Ox-42 IHC, in the injured hemisphere was significantly (*P* < 0.01) more important in QA (71.0 ± 9.8%), DFX-QA (48.7 ± 16.8%), DFX-H-QA (105.2 ± 11.5%), and in H-QA (542.8 ± 32.3%) than in both control and H groups (Figures 4(b) and 4(c)). The microglial activation measured in the H-QA group was significantly higher (*P* < 0.01) than in the QA, DFX-QA, and DFX-H-QA groups. Interestingly, the microglia activation level in DFX-QA, and QA groups was similar but DFX significantly decreased this activation in rats exposed to hemin treatment (*P* < 0.01 between DFX-QA and DFX-H-QA). 

### 3.3. Effect of Hemin Treatment and Influence of Ferrous Iron Chelator DFX on Neuronal Loss and ROS Production in the Striatum 1 h after Surgery

**Figure 5:**
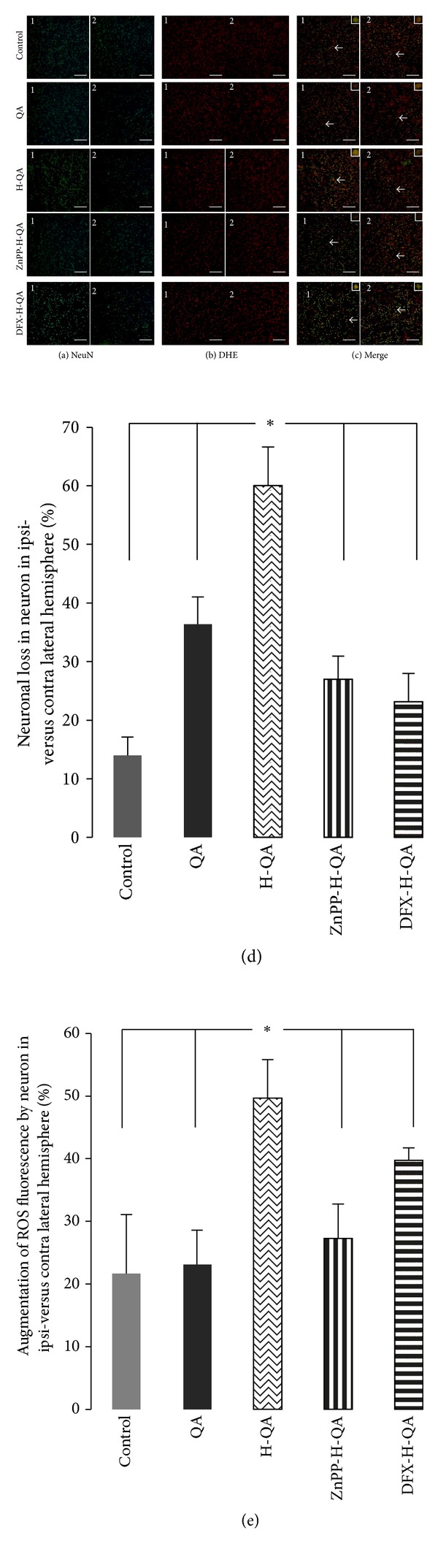
Immunofluorescence of NeuN and ethidium in rat striatum 1 h after QA injection. (a), (b), and (c) represent the same areas of contra- and ipsi-lateral striatum (1 and 2, resp.) of control (*n* = 5), QA (*n* = 5), H-QA (*n* = 5), ZnPP-H-QA (*n* = 5), and DFX-H-QA (*n* = 3) groups. Magnification was ×20 (×120 for inserts). Data are means  ±  SEM. Bars: 20 *μ*m. (a) NeuN (green channel). Neurons staining in contra- and ipsi-lateral striatum (1 and 2) for each group. Data are expressed as relative neuronal loss in the ipsilateral hemisphere versus the contralateral hemisphere (d). *P < 0.05 compared to respective contralateral side. (b) ROS expression analyzed with dihydroethidium (DHE) in the contra- and ipsi-lateral striatum (1 and 2) for each group. ROS measurements were performed at the same location as NeuN counting. Data are expressed as relative augmentation of ROS level in neurons in the ipsilateral hemisphere versus the contralateral hemisphere (e). *P < 0.05. (c) Merge representing neurons and ROS expression in contra- and ipsi-lateral striatum (1 and 2) for each group.

Intracellular ROS production was measured 1 h after QA injection with the DHE fluorometric method coupled with immunofluorescence NeuN-FITC neuron survival quantification to calculate ROS expression per neuron. Both measurements were performed on the same 3 areas for each hemisphere (3 slices per brain). Neuronal loss and ROS activity level were measured in control (*n* = 5), in QA (*n* = 5), in H-QA (*n* = 5), in DFX-H-QA (*n* = 3), and in ZnPP-H-QA (*n* = 5) rats exposed to chronic treatment with the inhibitor of HO-1 activity zinc protoporphyrin IX (Figures 5(a) and 5(b), resp.). Merge of neurons and ROS expression is represented in Figure 5(c).

After visual counting, the percentage of neuronal loss in the ipsilateral versus the contralateral hemispheres was analyzed. Neuronal loss in the ipsilateral hemisphere 1 h after surgery was significantly different between the control (14.0 ± 3.1%) and all other groups. Hemin significantly (*P* < 0.05) enhanced neuronal loss (60.0 ± 6.6% in H-QA group) when compared to QA alone (36.3 ± 4.6%). Both ZnPP and DFX limited the enhancement of this neuronal loss: 27.0 ± 3.9% and 23.1 ± 4.8% for ZnPP-H-QA and DFX-H-QA groups, respectively. These results are presented in Figure 5(d).

The percentage of augmentation of ROS level in neurons in the injured versus the contralateral striatum was significantly (*P* < 0.05) increased in the H-QA group (49  ±  6.2%) versus all the other groups. However, it is noteworthy that no significant difference was observed between control (21.7  ±  9.4%), QA (23.1  ±  5.5%), ZnPP-H-QA (27.3  ±  5.5%), and DFX-H-QA (39.7  ±  2.0%), although ROS increasing is greater in the latter group that in the others. Results of ROS production are summarized in Figure 5(e).

## 4. Discussion

We report here the effects of a chronic treatment with the direct HO-1 inductor and substrata hemin in an *in vivo* rodent model of excitotoxic neuroinflammation based on striatal quinolinic acid (QA) injection previously validated by our team [19]. Moreover, 3 days after QA injection was the time at which neuroinflammation was found to be at its greatest by our team using TSPO measurement (unpublished data) and has been chosen to analyze neuroinflammation in this study. The first step was to determine the optimal concentration of hemin that was able to significantly induce the expression of the HO-1 protein. Results obtained by WB showed that 4-day I.P. of hemin injections at a dose of 50 mg/kg were able to induce cerebral HO-1 expression significantly higher than in the control group or at a dose of 10 mg/kg of hemin. 

In the past decade, many studies have reported that HO-1 induction could be either beneficial or deleterious in the case of neuroinflammation, according to the *in vivo* or *in vitro* models and to the HO-1 induction procedure [32–34]. In our experimental conditions, the IHC method demonstrated the deleterious impact of the HO-1 induction, characterized by a significant lesion worsening in animals receiving hemin (H-QA group) versus QA rats. Therefore, we focused on the potential mechanism that could explain the harmful effect of HO-1 induction on neuroinflammation.

Microglial cells are the sensors of brain integrity through surface molecules, such as Toll-like or scavenger receptors, sensitive to background modifications that induce microglia activation [35]. In chronic neuroinflammation, unregulated microglia activity is responsible for neurotoxic factor production like ROS [5] that can lead to neuronal death. This has also been confirmed and quantified in our study where massive brain tissue destruction occurred 3 days after QA injection, amplified by hemin treatment (H-QA group). Since a brief time lapse suggested a fast and highly toxic destruction mechanism, we hypothesized an involvement of ROS in the cerebral tissue loss especially in this group. Because of their brief lifetime, ROS production has been measured 1 h after QA striatal injection by using the dihydroethidium (DHE) method that allows ROS detection only in intact cells. DHE penetrates into the cell where superoxide ion turns it into ethidium bromide which enters the cell nucleus and then inserts into the DNA before emitting a red fluorescence [36]. As the neuronal death was massive at the time of analysis, a double labeling was performed using NeuN coupled to FITC to have fluorescence related to the surviving neuron population. ROS fluorescence rationalized per neuron and compared in ipsi- versus contra-lateral striatum showed no difference between the QA and control groups, demonstrating that QA alone had no significant influence on ROS production. Conversely, chronic hemin treatment significantly promoted ROS production in the injured brain hemisphere in comparison to all other groups. The ROS production increasing in the H-QA group was inhibited in rats receiving the specific HO-1 inhibitor ZnPP, supporting the hypothesis that ROS level augmentation in hemin animals was a direct consequence of HO-1 activation. However, although a reduction of ROS production was observed in DFX-H-QA group, the ROS level remained higher than in the QA or ZnPP groups, suggesting the involvement of another mechanism than iron on ROS production. 

Induction of HO-1 results in the catabolism of the prooxidant heme to bile pigments, biliverdin, and bilirubin, which are potent antioxidants [37]. However, HO-1 metabolizes heme into free irons protoporphyrin then releases equimolar amounts of Fe^2+^ [38]. Free iron is well known for its catalyzing reaction that generates ROS and Schipper et al. [39] suggested that the negative effect of HO-1 induction observed in cultures of primary neonatal rat astroglia could be related to iron accumulation. Our results support this hypothesis in an *in vivo* animal model since the DFX iron chelator reduced tissue losses induced by the hemin treatment. Incidentally, it is to be noted that DFX had no effect on tissue destruction in animals receiving QA alone. As no difference was observed between controls and unexposed-to-QA hemin-treated rats (group H), we first hypothesized that QA and hemin developed a synergic destruction pattern. Considering the severity of the striatum lesions and those in the immediate surrounding areas, the microglial activation level measurement was performed in the cortical regions at a distance of 3 mm posterior to the site of injury. The microglia cells at J3 postlesion exhibited thick ramifications matching with activated microglia cells according to Kreutzberg [3]. However, no confusion with infiltrated macrophages could be made in our microscopic analysis as we only identified resting morphology cells in control rats and activated with ramifications microglia cells in H-QA rats. Results showed that microglia was significantly activated in QA animals versus the control in the ipsilateral hemisphere. It is noteworthy that this neuroinflammation was strongly enhanced by hemin in H-QA rats then matching with the tissue loss described. DFX treatment in DFX-H-QA group significantly decreased microglia activation in the ipsilateral hemisphere, strongly suggesting that Fe^2+^ may be implicated in deleterious HO-1 effects. Deleterious effects of microglia activation characterized by proinflammatory cytokines production (e.g., IL-1*β*, TNF*α*, iNOS) in striatum area have already been reported at J1 in this QA adult rat model [40]. Unfortunately, the massive tissue destruction observed in H-QA rats in our experimental conditions (J3 after QA injection) did not allow us to quantify the expression level of cytokines. The strong activation of microglia (Ox-42) measured at distance from the lesion (bregma −3 mm) in H-QA animals (Figure 4) was not associated with significant neuronal loss and no difference in neuronal survival in the cortex between the groups was observed. Indeed, the microglia activation in cortical area is not systematically associated with neurons death like in adult rats exposed to temporary brain ischemia [41]. Interestingly, the global level of activated microglia in this area was markedly more important in H versus control animals, suggesting a potential proinflammatory effect of HO-1 induction.

Therefore, we report here that HO-1 induction, ROS production, and cerebral destruction are closely linked in our neuroinflammatory model. ROS production itself could be directly connected to the HO-1 enzyme activity and probably results from a massive release of iron. The striatal injection of QA was responsible for tissue destruction but the induced excitotoxicity did not significantly modify ROS production 1 h after the injection. Nevertheless, hyperactivation of NMDA receptor is associated to massive intracellular Ca^2+^ entrance and accumulation leading to mitochondrial damages and nitric oxide synthase activation which are both responsible for ROS production [20]. So, we thought that the delay between QA injection and ROS production relating to QA effects take longer than one hour before reaching a significant level. 

The deleterious effect of HO-1 in this neuroinflammatory model could be explained by a hyperproduction of ROS. The diminution of both tissue loss and ROS production observed in rats receiving both DFX and hemin shows that iron is directly implicated in ROS production caused by HO-1 expression. Given that hemin alone did not induce either microglia activation or detectable neuronal death, it appears that the ROS-HO-1-mediated production created a cerebral destruction pattern in combination with the QA cerebral excitotoxicity. Constitutive heme oxygenase (HO-2) is also expressed in the brain and inhibited by ZnPP [42]. Its involvement in the deleterious effect associated to HO-1 activity cannot be totally rejected. HO-2 is mostly described for its role in the homeostasis of heme in brain [43] and therefore could participate in HO-1 regulation [44]. However, the specific influence of HO-2 in our model was not evaluated. 

Recently, it has been proposed that hemin caused microglia to release deleterious inflammatory factors via Toll-like receptor (TLR) 4 in a mouse model of intracerebral hemorrhage [45]. Indeed, exogenous hemin administration significantly increased microglial activation and exacerbated brain injury in WT mice but not in TLR4^−/−^  mice. Moreover, application of TLR4 antibody suppressed hemin-induced microglial activation in WT mice, suggesting a direct correlation between TLR4 and hemin-induced microglial activation. This was supported by the observation that TLR4 activates NF-*κ*B, which plays a critical role in the inflammatory response by regulating the gene expression of inflammatory mediators such as the cytokines IL-1*α* and-1*β*, TNF-*α*, and IL-6 [46, 47]. Therefore, targeting the TLR4 signaling pathway using anti-TLR4 antibody administration could be a potential therapeutic strategy in our excitotoxic rat model of neuroinflammation.

Complementary experiments could be carried out to determine the optimal hemin doses and administration schedule to achieve a positive benefit-risk balance of the drug. Indeed, most drugs of the Pharmacopoeia can generate deleterious effects if they are not used properly. With the aim of *in vivo *neuroprotection, the drug hemin should be associated to an ROS scavenger or an iron chelator. Thus, HO-1 activity promotes sequestration of redox-active iron in astroglia. This is confirmed by a recent review showing that selective human HO-1 expression in the astrocytes of transgenic mice is associated with iron sequestration in these cells [48]. Glial HO-1 may be a rational therapeutic target in AD, PD, and other human CNS conditions characterized by the unregulated deposition of brain iron.

In conclusion, this study showed first that hemin treatment and HO-1 induction had a deleterious effect in this QA model and enhanced tissue loss and microglia activation. Secondly, we showed that this effect is probably linked to a hyperproduction of ROS and iron accumulation. 

## References

[1] Glass CK, Saijo K, Winner B, Marchetto MC, Gage FH (2010). Mechanisms underlying inflammation in neurodegeneration. *Cell*.

[2] Ulvestad E, Williams K, Bjerkvig R, Tiekotter K, Antel J, Matre R (1994). Human microglial cells have phenotypic and functional characteristics in common with both macrophages and dendritic antigen-presenting cells. *Journal of Leukocyte Biology*.

[3] Kreutzberg GW (1996). Microglia: a sensor for pathological events in the CNS. *Trends in Neurosciences*.

[4] Ransohoff RM, Perry VH (2009). Microglial physiology: unique stimuli, specialized responses. *Annual Review of Immunology*.

[5] Block ML, Hong JS (2005). Microglia and inflammation-mediated neurodegeneration: multiple triggers with a common mechanism. *Progress in Neurobiology*.

[6] McGeer PL, McGeer EG (2007). NSAIDs and Alzheimer disease: epidemiological, animal model and clinical studies. *Neurobiology of Aging*.

[7] Gupta A, Kumar A, Kulkarni SK (2011). Targeting oxidative stress, mitochondrial dysfunction and neuroinflammatory signaling by selective cyclooxygenase (COX)-2 inhibitors mitigates MPTP-induced neurotoxicity in mice. *Progress in Neuro-Psychopharmacology and Biological Psychiatry*.

[8] Mirshafiey A, Matsuo H, Nakane S, Rehm BHA, Koh CS, Miyoshi S (2005). Novel immunosuppressive therapy by M2000 in experimental multiple sclerosis. *Immunopharmacology and Immunotoxicology*.

[9] Jantzen PT, Connor KE, DiCarlo G (2002). Microglial activation and *β*-amyloid deposit reduction caused by a nitric oxide-releasing nonsteroidal anti-inflammatory drug in amyloid precursor protein plus presenilin-1 transgenic mice. *Journal of Neuroscience*.

[10] Imbimbo BP (2009). An update on the efficacy of non-steroidal anti-inflammatory drugs in Alzheimer’s disease. *Expert Opinion on Investigational Drugs*.

[11] Tenhunen R, Marver HS, Schmid R (1968). The enzymatic conversion of heme to bilirubin by microsomal heme oxygenase. *Proceedings of the National Academy of Sciences of the United States of America*.

[12] Immenschuh S, Ramadori G (2000). Gene regulation of heme oxygenase-1 as a therapeutic target. *Biochemical Pharmacology*.

[13] Stocker R (2004). Antioxidant activities of bile pigments. *Antioxidants and Redox Signaling*.

[14] Jeong GS, Lee DS, Kim DC (2011). Neuroprotective and anti-inflammatory effects of mollugin via up-regulation of heme oxygenase-1 in mouse hippocampal and microglial cells. *European Journal of Pharmacology*.

[15] Chora A, Fontoura P, Cunha A (2007). Heme oxygenase-1 and carbon monoxide suppress autoimmune neuroinflammation. *Journal of Clinical Investigation*.

[16] Schipper HM (2000). Heme oxygenase-1: role in brain aging and neurodegeneration. *Experimental Gerontology*.

[17] Justicia C, Ramos-Cabrer P, Hoehn M (2008). MRI detection of secondary damage after stroke: chronic iron accumulation in the thalamus of the rat brain. *Stroke*.

[18] Schwarcz R, Köhler C (1983). Differential vulnerability of central neurons of the rat to quinolinic acid. *Neuroscience Letters*.

[19] Arlicot N, Katsifis A, Garreau L (2008). Evaluation of CLINDE as potent translocator protein (18 kDa) SPECT radiotracer reflecting the degree of neuroinflammation in a rat model of microglial activation. *European Journal of Nuclear Medicine and Molecular Imaging*.

[20] Estrada Sánchez AM, Mejía-Toiber J, Massieu L (2008). Excitotoxic neuronal death and the pathogenesis of Huntington's disease. *Archives of Medical Research*.

[21] Desbuards N, Rochefort GY, Schlecht D (2007). Heme oxygenase-1 inducer hemin prevents vascular thrombosis. *Thrombosis and Haemostasis*.

[22] Bektaşoğlu B, Esin Celik S, Ozyürek M, Güçlü K, Apak R (2006). Novel hydroxyl radical scavenging antioxidant activity assay for water-soluble antioxidants using a modified CUPRAC method. *Biochemical and Biophysical Research Communications*.

[23] Chang CK, Albarillo MV, Schumer W (2001). Therapeutic effect of dimethyl sulfoxide on ICAM-1 gene expression and activation of NF-*κ*B and AP-1 in septic rats. *Journal of Surgical Research*.

[24] Paxinos G, Watson C (1986). *The Rat Brain in Stereotaxic Coordonates*.

[25] Desbuards N, Hyvelin JM, Machet MC (2009). Heme oxygenase-1 inducer hemin attenuates the progression of remnant kidney model. *Nephron—Experimental Nephrology*.

[26] Borgmann S (2009). Electrochemical quantification of reactive oxygen and nitrogen: challenges and opportunities. *Analytical and Bioanalytical Chemistry*.

[27] Kalyanaraman B (2011). Oxidative chemistry of fluorescent dyes: implications in the detection of reactive oxygen and nitrogen species. *Biochemical Society Transactions*.

[28] Khandelwal S, Saxena RK (2007). Age-dependent increase in green autofluorescence of blood erythrocytes. *Journal of Biosciences*.

[29] Poulet R, Gentile MT, Vecchione C (2006). Acute hypertension induces oxidative stress in brain tissues. *Journal of Cerebral Blood Flow and Metabolism*.

[30] Shichinohe H, Kuroda S, Yasuda H (2004). Neuroprotective effects of the free radical scavenger Edaravone (MCI-186) in mice permanent focal brain ischemia. *Brain Research*.

[31] Yamamoto E, Tamamaki N, Nakamura T (2008). Excess salt causes cerebral neuronal apoptosis and inflammation in stroke-prone hypertensive rats through angiotensin II-induced NADPH oxidase activation. *Stroke*.

[32] Lu DY, Tsao YY, Leung YM, Su KP (2010). Docosahexaenoic acid suppresses neuroinflammatory responses and induces heme oxygenase-1 expression in BV-2 microglia: implications of antidepressant effects for omega-3 fatty acids. *Neuropsychopharmacology*.

[33] Laird MD, Wakade C, Alleyne CH, Dhandapani KM (2008). Hemin-induced necroptosis involves glutathione depletion in mouse astrocytes. *Free Radical Biology and Medicine*.

[34] Park JS, Shin JA, Jung JS (2012). Anti-inflammatory mechanism of compound K in activated microglia and its neuroprotective effect on experimental stroke in mice. *Journal of Pharmacology and Experimental Therapeutics*.

[35] Kettenmann H, Hanisch UK, Noda M, Verkhratsky A (2011). Physiology of microglia. *Physiological Reviews*.

[36] Zhao H, Joseph J, Fales HM (2005). Detection and characterization of the product of hydroethidine and intracellular superoxide by HPLC and limitations of fluorescence. *Proceedings of the National Academy of Sciences of the United States of America*.

[37] Morita T (2005). Heme oxygenase and atherosclerosis. *Arteriosclerosis, Thrombosis, and Vascular Biology*.

[38] Syapin PJ (2008). Regulation of haeme oxygenase-1 for treatment of neuroinflammation and brain disorders. *British Journal of Pharmacology*.

[39] Schipper HM, Gupta A, Szarek WA (2009). Suppression of glial HO-1 activitiy as a potential neurotherapeutic intervention in AD. *Current Alzheimer Research*.

[40] Ryu JK, Choi HB, McLarnon JG (2005). Peripheral benzodiazepine receptor ligand PK11195 reduces microglial activation and neuronal death in quinolinic acid-injected rat striatum. *Neurobiology of Disease*.

[41] Soltys Z, Orzylowska-Sliwinska O, Zaremba M (2005). Quantitative morphological study of microglial cells in the ischemic rat brain using principal component analysis. *Journal of Neuroscience Methods*.

[42] Wong RJ, Vreman HJ, Schulz S, Kalish FS, Pierce NW, Stevenson DK (2011). In vitro inhibition of heme oxygenase isoenzymes by metalloporphyrins. *Journal of Perinatology*.

[43] Kim YM, Pae HO, Park JE (2011). Heme oxygenase in the regulation of vascular biology: from molecular mechanisms to therapeutic opportunities. *Antioxidants & Redox Signaling*.

[44] Sodhi K, Inoue K, Gotlinger KH (2009). Epoxyeicosatrienoic acid agonist rescues the metabolic syndrome phenotype of HO-2-null mice. *Journal of Pharmacology and Experimental Therapeutics*.

[45] Lin S, Zhong Q, Lv FL (2012). Heme activates TLR4-mediated inflammatory injury via MyD88/TRIF signaling pathway in intracerebral hemorrhage. *Journal of Neuroinflammation*.

[46] Miyake K (2004). Endotoxin recognition molecules MD-2 and toll-like receptor 4 as potential targets for therapeutic intervention of endotoxin shock. *Current Drug Targets: Inflammation and Allergy*.

[47] Takeda K, Akira S (2004). TLR signaling pathways. *Seminars in Immunology*.

[48] Schipper HM (2011). Heme oxygenase-1 in Alzheimer disease: a tribute to Moussa Youdim. *Journal of Neural Transmission*.

